# 4-Formyl­phenyl 2,3,4,6-tetra-*O*-acetyl-β-d-allopyran­oside

**DOI:** 10.1107/S1600536809018248

**Published:** 2009-05-20

**Authors:** Ding Ye, Kuan Zhang, Hua-feng Chen, Shu-fan Yin, Ying Li

**Affiliations:** a College of Chemistry, Sichuan University, Chengdu 610064, People’s Republic of China

## Abstract

The title compound, C_21_H_24_O_11_, crystallizes exclusively as the β-anomer. The substituent of the protected sugar at position C-3 is in the axial position, while all other groups are in equatorial positions. The pyran­oside ring adopts a stable chair conformation.

## Related literature

For the synthesis see: Chen *et al.* (1981 ▶); Wen *et al.* (2008 ▶). For the pharmacological activities of helicid derivatives, see: Fan *et al.* (2008 ▶), Sha *et al.* (1987 ▶). For related structures, see: Burkhardt *et al.* (2007*a* ▶, 2007*b* ▶)
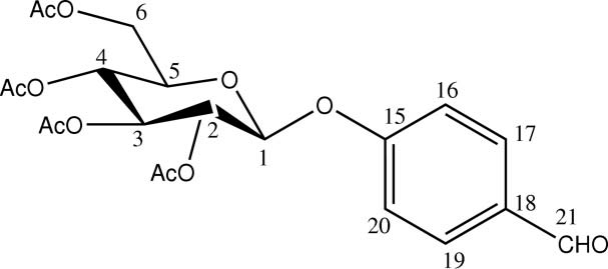


## Experimental

### 

#### Crystal data

C_21_H_24_O_11_
*M* *_r_* = 452.40
Monoclinic, 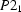

*a* = 7.056 (4) Å
*b* = 17.758 (6) Å
*c* = 9.129 (3) Å
β = 102.80 (4)°
*V* = 1115.4 (8) Å^3^
*Z* = 2
Mo *K*α radiation
μ = 0.11 mm^−1^
*T* = 291 K
0.44 × 0.42 × 0.20 mm

#### Data collection

Enraf–Nonius CAD-4 diffractometer
Absorption correction: none
4596 measured reflections
4149 independent reflections
2143 reflections with *I* > 2σ(*I*)
*R*_int_ = 0.029
3 standard reflections every 150 reflections intensity decay: 3.7%

#### Refinement

*R*[*F* ^2^ > 2σ(*F* ^2^)] = 0.050
*wR*(*F* ^2^) = 0.143
*S* = 0.95
4149 reflections
293 parameters
1 restraint
H-atom parameters constrained
Δρ_max_ = 0.30 e Å^−3^
Δρ_min_ = −0.20 e Å^−3^

### 

Data collection: *DIFRAC* (Gabe & White, 1993 ▶); cell refinement: *DIFRAC*; data reduction: *NRCVAX* (Gabe *et al.*, 1989 ▶); program(s) used to solve structure: *SHELXS97* (Sheldrick, 2008 ▶); program(s) used to refine structure: *SHELXL97* (Sheldrick, 2008 ▶); molecular graphics: *ORTEP-3 for Windows* (Farrugia, 1997 ▶); software used to prepare material for publication: *SHELXL97*.

## Supplementary Material

Crystal structure: contains datablocks global, I. DOI: 10.1107/S1600536809018248/bx2200sup1.cif

Structure factors: contains datablocks I. DOI: 10.1107/S1600536809018248/bx2200Isup2.hkl

Additional supplementary materials:  crystallographic information; 3D view; checkCIF report

## Figures and Tables

**Fig. 1.**
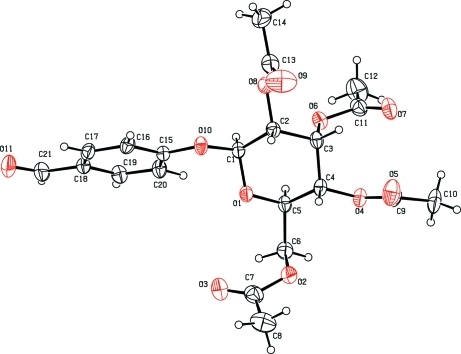
The molecular structure of (I), with displacement ellipsoids drawn at the 30% probability level.

## supplementary crystallographic information

### Comment

The natural compound helicid, 4-(β-D-allopyranosyloxy)benzaldehyde (Chen *et al.*, 1981), is a major active ingredient of Chinese herbal medicine, which has good biological effects on central nervous system with low toxicity (Sha *et al.*, 1987). Some helicid derivatives have been reported with good pharmacological activities (Fan *et al.*, 2008). The title compound, a new helicid derivative, was synthesized *via* reaction of helicid and acetyl anhydride with good yield of 98% (Wen *et al.*, 2008). Herein, we describe the structure of 4-formylphenyl-2,3, 4,6-tetra-*O*-acetyl-β-D-allopyranoside which compare well with the related structures 3-Formylphenyl-2,3,4,6-tetra-*O*-acetyl-β-D– glucopyranoside (Burkhardt *et al.*, 2007a) and 3-Formylphenyl-2,3,4,6-tetra-*O*-acetyl-α -D– glucopyranoside (Burkhardt *et al.*, 2007b) . The 4-formylphenyl group is subsituted at anomeric atom C1. The remaining hydroxy groups at C2, C3, C4 and C6 are protected by acetyl groups. Due to its hydrophobic substituents the compound is soluble in less polar solvents such as CH_2_Cl_2_. The 4-formylphenyl substituent at C1 is in an equatorial position, corresponding to the exclusive presence of the β anomer of the saccharide.The substituent of the protected sugar at C3 is in the axial position.

### Experimental

To a solution of helicid (1.0 g, 3.5 mmol) in 2 ml of DMF and 3 ml of TEA was added dropwise acetyl anhydride (2.5 g, 25 mmol) under ice bath. The mixture was stirred vigorously at room temperature 5 h, and then poured into 20 ml of ice water. The precipitate was filtered, washed with water, and recrystallized with ethanol. By slow evaporation at room temperature, we got colorless crystals, yield 98%, m.p.:408–409 K. IR (KBr): 1752, 1693, 1601, 1506, 1224, 1157, 1127, 1085, 914, 876; ^1^H NMR (400 MHz, CDCl_3_, 298 K): 2.18, 2.08, 2.05, 2.05 (4 s, 12H, CH3); 4.26–4.24 (m, 2H, H6a, H6e); 4.32–4.27 (m, 1H, H5); 5.16 (dd, 1H, J_43_ = 2.8 Hz, J_45_ = 9.9 Hz, H4); 5.19 (dd, 1H, J_23_ = 3.0 Hz, J_21_ = 8.1 Hz, H2); 5.48 (d, 1H, J_12_ = 8.1 Hz, H1); 5.75 (t, 1H, J_32_ = 2.9 Hz, H3); 7.85 (d, 2H, J_89_ = J_1211_ = 8.6 Hz, ArH); 7.13 (d, 2H, J_910_ = J_1110_ = 8.6 Hz, ArH); 9.91 (s, 1H, H—C=O) p.p.m. ^13^C NMR (100 MHz, CDCl_3_, 298 K): 191.2, 170.8, 170.6, 170.5, 169.8, 161.7, 132.3, 117.2, 99.0, 71.8, 71.1, 68.8, 67.2, 61.8, 21.2, 21.1,21.0 p.p.m. ESI-MS: m/*z* (%) = 475 *[M + Na]*^+^ (100). Analysis calculated for C_21_H_24_O_11_.

### Refinement

H atoms were positioned geometrically (C—H = 0.93–0.98 Å) and refined using a riding model, with *U*_iso_(H) = 1.2*U*_eq_ (methylene C, aromatic C), *U*_iso_(H) = 1.5*U*_eq_ (methyl C).

### Crystal data

**Table:**

C_21_H_24_O_11_	*F*(000) = 476
*M**_r_* = 452.40	*D*_x_ = 1.347 Mg m^−^^3^
Monoclinic, *P*2_1_	Mo *K*α radiation, λ = 0.71073 Å
Hall symbol: P 2yb	Cell parameters from 33 reflections
*a* = 7.056 (4) Å	θ = 4.6–9.4°
*b* = 17.758 (6) Å	µ = 0.11 mm^−^^1^
*c* = 9.129 (3) Å	*T* = 291 K
β = 102.80 (4)°	Block, colourless
*V* = 1115.4 (8) Å^3^	0.44 × 0.42 × 0.20 mm
*Z* = 2	

### Data collection

**Table:**

Enraf–Nonius CAD-4 diffractometer	*R*_int_ = 0.029
Radiation source: fine-focus sealed tube	θ_max_ = 32.5°, θ_min_ = 2.3°
graphite	*h* = −10→10
ω/2θ scans	*k* = −25→26
4596 measured reflections	*l* = −13→13
4149 independent reflections	3 standard reflections every 150 reflections
2143 reflections with *I* > 2σ(*I*)	intensity decay: 3.7%

### Refinement

**Table:**

Refinement on *F*^2^	Primary atom site location: structure-invariant direct methods
Least-squares matrix: full	Secondary atom site location: difference Fourier map
*R*[*F*^2^ > 2σ(*F*^2^)] = 0.050	Hydrogen site location: inferred from neighbouring sites
*wR*(*F*^2^) = 0.143	H-atom parameters constrained
*S* = 0.95	*w* = 1/[σ^2^(*F*_o_^2^) + (0.0778*P*)^2^] where *P* = (*F*_o_^2^ + 2*F*_c_^2^)/3
4149 reflections	(Δ/σ)_max_ < 0.001
293 parameters	Δρ_max_ = 0.30 e Å^−^^3^
1 restraint	Δρ_min_ = −0.20 e Å^−^^3^

### Special details

**Table:**

Geometry. All e.s.d.'s (except the e.s.d. in the dihedral angle between two l.s. planes) are estimated using the full covariance matrix. The cell e.s.d.'s are taken into account individually in the estimation of e.s.d.'s in distances, angles and torsion angles; correlations between e.s.d.'s in cell parameters are only used when they are defined by crystal symmetry. An approximate (isotropic) treatment of cell e.s.d.'s is used for estimating e.s.d.'s involving l.s. planes.
Refinement. Refinement of *F*^2^ against ALL reflections. The weighted *R*-factor *wR* and goodness of fit *S* are based on *F*^2^, conventional *R*-factors *R* are based on *F*, with *F* set to zero for negative *F*^2^. The threshold expression of *F*^2^ > σ(*F*^2^) is used only for calculating *R*-factors(gt) *etc*. and is not relevant to the choice of reflections for refinement. *R*-factors based on *F*^2^ are statistically about twice as large as those based on *F*, and *R*- factors based on ALL data will be even larger.

### Fractional atomic coordinates and isotropic or equivalent isotropic displacement parameters (Å^2^)

**Table:**

	*x*	*y*	*z*	*U*_iso_*/*U*_eq_	
O1	0.4950 (3)	0.63276 (10)	0.1617 (2)	0.0399 (4)	
O2	0.2064 (3)	0.55171 (14)	−0.0604 (2)	0.0526 (5)	
O3	0.3692 (5)	0.62506 (18)	−0.1903 (3)	0.0857 (9)	
O4	0.2271 (3)	0.48018 (11)	0.2871 (2)	0.0436 (4)	
O5	−0.0540 (3)	0.52455 (15)	0.3289 (4)	0.0733 (8)	
O6	0.5256 (3)	0.54679 (12)	0.5011 (2)	0.0446 (4)	
O7	0.3283 (4)	0.48347 (14)	0.6212 (3)	0.0657 (7)	
O8	0.5465 (3)	0.69998 (12)	0.5428 (2)	0.0468 (5)	
O9	0.2909 (5)	0.7687 (2)	0.5597 (4)	0.1025 (12)	
O10	0.6171 (3)	0.74339 (10)	0.2628 (2)	0.0415 (4)	
O11	1.2834 (4)	0.88786 (18)	−0.0222 (3)	0.0780 (8)	
C1	0.5854 (4)	0.66770 (14)	0.2979 (3)	0.0370 (5)	
H1	0.7070	0.6424	0.3454	0.044*	
C2	0.4428 (4)	0.67009 (15)	0.4018 (3)	0.0385 (6)	
H2	0.3343	0.7036	0.3589	0.046*	
C3	0.3648 (4)	0.59176 (16)	0.4229 (3)	0.0394 (6)	
H3	0.2627	0.5944	0.4801	0.047*	
C4	0.2873 (4)	0.55657 (15)	0.2697 (3)	0.0388 (6)	
H4	0.1757	0.5858	0.2156	0.047*	
C5	0.4441 (4)	0.55511 (15)	0.1781 (3)	0.0382 (6)	
H5	0.5581	0.5283	0.2352	0.046*	
C6	0.3858 (4)	0.52131 (17)	0.0245 (3)	0.0482 (7)	
H6A	0.4876	0.5304	−0.0293	0.058*	
H6B	0.3723	0.4673	0.0338	0.058*	
C7	0.2171 (6)	0.6017 (2)	−0.1706 (4)	0.0620 (9)	
C8	0.0234 (7)	0.6216 (3)	−0.2607 (5)	0.0897 (13)	
H8A	0.0274	0.6713	−0.3014	0.135*	
H8B	−0.0694	0.6204	−0.1981	0.135*	
H8C	−0.0141	0.5862	−0.3412	0.135*	
C9	0.0503 (4)	0.47285 (19)	0.3242 (4)	0.0528 (8)	
C10	0.0162 (6)	0.3927 (2)	0.3635 (5)	0.0743 (11)	
H10A	0.0886	0.3821	0.4634	0.111*	
H10B	0.0578	0.3596	0.2937	0.111*	
H10C	−0.1198	0.3851	0.3586	0.111*	
C11	0.4840 (5)	0.49291 (18)	0.5944 (3)	0.0535 (8)	
C12	0.6629 (7)	0.4484 (3)	0.6583 (6)	0.0856 (13)	
H12A	0.7167	0.4648	0.7591	0.128*	
H12B	0.7566	0.4557	0.5978	0.128*	
H12C	0.6303	0.3959	0.6590	0.128*	
C13	0.4540 (5)	0.75101 (18)	0.6110 (4)	0.0563 (8)	
C14	0.5769 (7)	0.7780 (3)	0.7549 (5)	0.0842 (13)	
H14A	0.5345	0.8273	0.7771	0.126*	
H14B	0.7100	0.7804	0.7465	0.126*	
H14C	0.5656	0.7439	0.8341	0.126*	
C15	0.7685 (4)	0.75945 (14)	0.1960 (3)	0.0359 (5)	
C16	0.8011 (4)	0.83541 (16)	0.1761 (3)	0.0430 (6)	
H16	0.7223	0.8714	0.2069	0.052*	
C17	0.9503 (4)	0.85760 (16)	0.1108 (3)	0.0453 (7)	
H17	0.9704	0.9085	0.0961	0.054*	
C18	1.0703 (4)	0.80472 (17)	0.0669 (3)	0.0417 (6)	
C19	1.0361 (4)	0.72875 (17)	0.0863 (3)	0.0460 (7)	
H19	1.1167	0.6930	0.0568	0.055*	
C20	0.8838 (4)	0.70516 (16)	0.1487 (3)	0.0443 (6)	
H20	0.8594	0.6542	0.1587	0.053*	
C21	1.2373 (5)	0.8255 (2)	0.0018 (4)	0.0587 (8)	
H21	1.3126	0.7864	−0.0224	0.070*	

### Atomic displacement parameters (Å^2^)

**Table:**

	*U*^11^	*U*^22^	*U*^33^	*U*^12^	*U*^13^	*U*^23^
O1	0.0456 (10)	0.0342 (9)	0.0435 (10)	−0.0048 (8)	0.0178 (8)	0.0015 (8)
O2	0.0535 (12)	0.0559 (12)	0.0508 (11)	−0.0077 (10)	0.0171 (10)	−0.0018 (10)
O3	0.101 (2)	0.085 (2)	0.0768 (18)	−0.0286 (18)	0.0309 (15)	0.0115 (15)
O4	0.0422 (10)	0.0337 (10)	0.0598 (11)	−0.0029 (8)	0.0218 (9)	0.0005 (9)
O5	0.0492 (13)	0.0593 (16)	0.121 (2)	0.0022 (12)	0.0392 (14)	0.0042 (15)
O6	0.0462 (11)	0.0442 (10)	0.0462 (10)	0.0045 (9)	0.0162 (8)	0.0099 (8)
O7	0.0768 (17)	0.0592 (15)	0.0691 (15)	−0.0078 (13)	0.0330 (13)	0.0151 (13)
O8	0.0454 (11)	0.0481 (11)	0.0502 (11)	−0.0016 (9)	0.0177 (9)	−0.0086 (9)
O9	0.102 (2)	0.109 (3)	0.097 (2)	0.049 (2)	0.0209 (18)	−0.0315 (19)
O10	0.0459 (10)	0.0294 (9)	0.0559 (11)	0.0004 (8)	0.0257 (9)	0.0009 (8)
O11	0.0760 (18)	0.0810 (19)	0.0855 (19)	−0.0344 (15)	0.0361 (15)	−0.0012 (15)
C1	0.0399 (13)	0.0320 (12)	0.0432 (13)	−0.0019 (10)	0.0176 (11)	0.0001 (11)
C2	0.0402 (14)	0.0360 (13)	0.0427 (13)	0.0008 (11)	0.0167 (11)	−0.0013 (11)
C3	0.0367 (13)	0.0387 (13)	0.0478 (14)	0.0010 (11)	0.0197 (11)	0.0035 (12)
C4	0.0374 (13)	0.0300 (12)	0.0521 (15)	−0.0012 (11)	0.0166 (11)	0.0027 (11)
C5	0.0399 (14)	0.0329 (13)	0.0465 (14)	−0.0001 (11)	0.0195 (11)	0.0010 (11)
C6	0.0526 (17)	0.0426 (15)	0.0540 (17)	−0.0005 (13)	0.0216 (14)	−0.0051 (13)
C7	0.086 (3)	0.0523 (19)	0.0521 (18)	−0.0007 (18)	0.0236 (18)	−0.0051 (15)
C8	0.099 (3)	0.102 (3)	0.063 (2)	0.017 (3)	0.006 (2)	0.004 (2)
C9	0.0444 (16)	0.0487 (18)	0.070 (2)	−0.0114 (15)	0.0227 (14)	−0.0075 (16)
C10	0.078 (3)	0.049 (2)	0.105 (3)	−0.0242 (19)	0.039 (2)	0.005 (2)
C11	0.068 (2)	0.0462 (17)	0.0481 (16)	0.0011 (15)	0.0168 (15)	0.0058 (14)
C12	0.089 (3)	0.079 (3)	0.088 (3)	0.020 (2)	0.018 (2)	0.032 (2)
C13	0.070 (2)	0.0432 (17)	0.0644 (19)	0.0026 (16)	0.0339 (17)	−0.0080 (15)
C14	0.096 (3)	0.090 (3)	0.077 (2)	−0.037 (3)	0.041 (2)	−0.036 (2)
C15	0.0365 (13)	0.0345 (13)	0.0379 (13)	−0.0017 (10)	0.0108 (10)	0.0020 (10)
C16	0.0446 (15)	0.0302 (13)	0.0559 (17)	−0.0023 (11)	0.0148 (13)	−0.0053 (12)
C17	0.0479 (16)	0.0354 (14)	0.0534 (16)	−0.0104 (12)	0.0132 (13)	−0.0016 (12)
C18	0.0395 (13)	0.0463 (16)	0.0408 (13)	−0.0058 (12)	0.0125 (11)	0.0022 (12)
C19	0.0489 (16)	0.0406 (15)	0.0530 (16)	0.0063 (13)	0.0210 (13)	0.0013 (12)
C20	0.0501 (15)	0.0312 (13)	0.0568 (16)	0.0019 (12)	0.0230 (13)	0.0011 (12)
C21	0.0525 (18)	0.070 (2)	0.0581 (18)	−0.0109 (17)	0.0212 (15)	0.0046 (17)

### Geometric parameters (Å, °)

**Table:**

O1—C1	1.409 (3)	C7—C8	1.474 (6)
O1—C5	1.441 (3)	C8—H8A	0.9600
O2—C7	1.356 (4)	C8—H8B	0.9600
O2—C6	1.435 (4)	C8—H8C	0.9600
O3—C7	1.201 (5)	C9—C10	1.500 (5)
O4—C9	1.369 (4)	C10—H10A	0.9600
O4—C4	1.441 (3)	C10—H10B	0.9600
O5—C9	1.184 (4)	C10—H10C	0.9600
O6—C11	1.356 (4)	C11—C12	1.494 (5)
O6—C3	1.441 (4)	C12—H12A	0.9600
O7—C11	1.189 (4)	C12—H12B	0.9600
O8—C13	1.347 (4)	C12—H12C	0.9600
O8—C2	1.435 (3)	C13—C14	1.484 (5)
O9—C13	1.185 (5)	C14—H14A	0.9600
O10—C15	1.372 (3)	C14—H14B	0.9600
O10—C1	1.411 (3)	C14—H14C	0.9600
O11—C21	1.188 (5)	C15—C16	1.387 (4)
C1—C2	1.529 (3)	C15—C20	1.391 (4)
C1—H1	0.9800	C16—C17	1.377 (4)
C2—C3	1.524 (4)	C16—H16	0.9300
C2—H2	0.9800	C17—C18	1.382 (4)
C3—C4	1.519 (4)	C17—H17	0.9300
C3—H3	0.9800	C18—C19	1.389 (4)
C4—C5	1.528 (3)	C18—C21	1.479 (4)
C4—H4	0.9800	C19—C20	1.388 (4)
C5—C6	1.497 (4)	C19—H19	0.9300
C5—H5	0.9800	C20—H20	0.9300
C6—H6A	0.9700	C21—H21	0.9300
C6—H6B	0.9700		
			
C1—O1—C5	113.85 (19)	H8B—C8—H8C	109.5
C7—O2—C6	117.3 (3)	O5—C9—O4	122.9 (3)
C9—O4—C4	115.1 (2)	O5—C9—C10	126.4 (3)
C11—O6—C3	116.5 (2)	O4—C9—C10	110.7 (3)
C13—O8—C2	117.4 (2)	C9—C10—H10A	109.5
C15—O10—C1	118.5 (2)	C9—C10—H10B	109.5
O1—C1—O10	106.5 (2)	H10A—C10—H10B	109.5
O1—C1—C2	109.2 (2)	C9—C10—H10C	109.5
O10—C1—C2	105.9 (2)	H10A—C10—H10C	109.5
O1—C1—H1	111.7	H10B—C10—H10C	109.5
O10—C1—H1	111.7	O7—C11—O6	124.5 (3)
C2—C1—H1	111.7	O7—C11—C12	125.8 (3)
O8—C2—C3	110.5 (2)	O6—C11—C12	109.7 (3)
O8—C2—C1	107.0 (2)	C11—C12—H12A	109.5
C3—C2—C1	111.0 (2)	C11—C12—H12B	109.5
O8—C2—H2	109.4	H12A—C12—H12B	109.5
C3—C2—H2	109.4	C11—C12—H12C	109.5
C1—C2—H2	109.4	H12A—C12—H12C	109.5
O6—C3—C4	108.2 (2)	H12B—C12—H12C	109.5
O6—C3—C2	107.6 (2)	O9—C13—O8	121.5 (3)
C4—C3—C2	109.0 (2)	O9—C13—C14	126.2 (3)
O6—C3—H3	110.7	O8—C13—C14	112.2 (3)
C4—C3—H3	110.7	C13—C14—H14A	109.5
C2—C3—H3	110.7	C13—C14—H14B	109.5
O4—C4—C3	109.9 (2)	H14A—C14—H14B	109.5
O4—C4—C5	108.1 (2)	C13—C14—H14C	109.5
C3—C4—C5	110.7 (2)	H14A—C14—H14C	109.5
O4—C4—H4	109.3	H14B—C14—H14C	109.5
C3—C4—H4	109.3	O10—C15—C16	115.4 (2)
C5—C4—H4	109.3	O10—C15—C20	124.1 (2)
O1—C5—C6	108.0 (2)	C16—C15—C20	120.5 (2)
O1—C5—C4	105.6 (2)	C17—C16—C15	120.0 (3)
C6—C5—C4	115.9 (2)	C17—C16—H16	120.0
O1—C5—H5	109.0	C15—C16—H16	120.0
C6—C5—H5	109.0	C16—C17—C18	120.5 (3)
C4—C5—H5	109.0	C16—C17—H17	119.8
O2—C6—C5	112.4 (2)	C18—C17—H17	119.8
O2—C6—H6A	109.1	C17—C18—C19	119.2 (3)
C5—C6—H6A	109.1	C17—C18—C21	122.7 (3)
O2—C6—H6B	109.1	C19—C18—C21	118.0 (3)
C5—C6—H6B	109.1	C20—C19—C18	121.2 (3)
H6A—C6—H6B	107.8	C20—C19—H19	119.4
O3—C7—O2	122.4 (4)	C18—C19—H19	119.4
O3—C7—C8	125.6 (4)	C19—C20—C15	118.6 (3)
O2—C7—C8	111.9 (4)	C19—C20—H20	120.7
C7—C8—H8A	109.5	C15—C20—H20	120.7
C7—C8—H8B	109.5	O11—C21—C18	125.6 (4)
H8A—C8—H8B	109.5	O11—C21—H21	117.2
C7—C8—H8C	109.5	C18—C21—H21	117.2
H8A—C8—H8C	109.5		
			
C5—O1—C1—O10	−177.44 (19)	C3—C4—C5—C6	179.7 (2)
C5—O1—C1—C2	−63.6 (3)	C7—O2—C6—C5	104.7 (3)
C15—O10—C1—O1	−76.9 (3)	O1—C5—C6—O2	−68.4 (3)
C15—O10—C1—C2	167.0 (2)	C4—C5—C6—O2	49.8 (3)
C13—O8—C2—C3	−101.7 (3)	C6—O2—C7—O3	−6.0 (5)
C13—O8—C2—C1	137.3 (2)	C6—O2—C7—C8	173.2 (3)
O1—C1—C2—O8	175.1 (2)	C4—O4—C9—O5	6.4 (5)
O10—C1—C2—O8	−70.7 (3)	C4—O4—C9—C10	−171.0 (3)
O1—C1—C2—C3	54.4 (3)	C3—O6—C11—O7	−4.2 (5)
O10—C1—C2—C3	168.6 (2)	C3—O6—C11—C12	176.3 (3)
C11—O6—C3—C4	−94.4 (3)	C2—O8—C13—O9	2.7 (5)
C11—O6—C3—C2	148.0 (2)	C2—O8—C13—C14	−179.5 (3)
O8—C2—C3—O6	−53.2 (3)	C1—O10—C15—C16	−174.9 (2)
C1—C2—C3—O6	65.4 (3)	C1—O10—C15—C20	5.6 (4)
O8—C2—C3—C4	−170.2 (2)	O10—C15—C16—C17	179.6 (2)
C1—C2—C3—C4	−51.7 (3)	C20—C15—C16—C17	−0.8 (4)
C9—O4—C4—C3	78.4 (3)	C15—C16—C17—C18	−1.1 (4)
C9—O4—C4—C5	−160.6 (2)	C16—C17—C18—C19	1.5 (4)
O6—C3—C4—O4	58.7 (2)	C16—C17—C18—C21	−177.5 (3)
C2—C3—C4—O4	175.4 (2)	C17—C18—C19—C20	0.0 (4)
O6—C3—C4—C5	−60.7 (3)	C21—C18—C19—C20	179.1 (3)
C2—C3—C4—C5	56.0 (3)	C18—C19—C20—C15	−1.9 (4)
C1—O1—C5—C6	−169.3 (2)	O10—C15—C20—C19	−178.2 (3)
C1—O1—C5—C4	66.1 (3)	C16—C15—C20—C19	2.3 (4)
O4—C4—C5—O1	178.8 (2)	C17—C18—C21—O11	−1.5 (5)
C3—C4—C5—O1	−60.7 (3)	C19—C18—C21—O11	179.4 (3)
O4—C4—C5—C6	59.3 (3)		
